# Transcriptome Analysis Suggests That Starch Synthesis May Proceed via Multiple Metabolic Routes in High Yielding Potato Cultivars

**DOI:** 10.1371/journal.pone.0051248

**Published:** 2012-12-17

**Authors:** Kacper Piotr Kaminski, Annabeth Høgh Petersen, Mads Sønderkær, Lars Haastrup Pedersen, Henrik Pedersen, Christian Feder, Kåre L. Nielsen

**Affiliations:** 1 Department of Agroecology, Aarhus University, Aarhus, Denmark; 2 Department of Biotechnology, Chemistry and Environmental Engineering, Aalborg University, Aalborg, Denmark; 3 AKV-Langholt Amba, Langholt, Denmark; 4 KMC, Brande, Denmark; University of North Carolina at Charlotte, United States of America

## Abstract

**Background:**

Glucose-6-phosphate is imported into the amyloplast of potato tubers and thought to constitute the precursor for starch synthesis in potato tubers. However, recently it was shown that glucose-1-phosphate can also be imported into the amyloplast and incorporated into starch via an ATP independent mechanism under special conditions. Nonetheless, glucose-6-phosphate is believed to be the quantitatively important precursor for starch synthesis in potato.

**Principal Finding:**

Potato tubers of the high yielding cv Kuras had low gene expression of plastidial phophoglucomutase (PGM) and normal levels of transcripts for other enzymes involved in starch metabolism in comparison with medium and low yielding cultivars as determined by DeepSAGE transcriptome profiling. The decrease in PGM activity in Kuras was confirmed by measuring the enzyme activity from potato tuber extracts. Contrary to expectations, this combination lead to a higher level of intracellular glucose-1-phosphate (G1P) in Kuras suggesting that G1P is directly imported into plastids and can be quantitatively important for starch synthesis under normal conditions in high yielding cultivars.

**Significance:**

This could open entirely new possibilities for metabolic engineering of the starch metabolism in potato via the so far uncharacterized G1P transporter. The perspectives are to increase yield and space efficiency of this important crop. In the light of the increasing demands imposed on agriculture to support a growing global population this presents an exciting new possibility.

## Introduction

Potato (*Solanum tuberosum*) is the fourth most important crop in the world and belongs to the *Solanaceae* family together with other important crops as tomato, pepper and tobacco. Potato is grown virtually all over the world with an overall production estimated to 329.6 million tonnes from 18.7 million hectares (www.faostat.fao.org, 2009) with China being the largest producer. In contrast to other major starch producing crops such as wheat, rice and maize, potatoes are grown for their starch-rich tubers formed from underground stems (stolons). Potato has therefore, like beets and cassava, the storage organ supported by the soil and cultivars with increased yield can be developed without concomitant increase in metabolic costs for support tissue robust enough to carry the increased weight of the storage organs. Arguably because of this, potatoes are very space efficient crops yielding roughly twice the number of calories per area unit as cereals and producing energy yields similar to sugar cane. Crops with the highest yield per area unit are becoming increasingly important because of the great demand for global crop yield increase to support a burgeoning growth of the world population and little opportunities to include previously unexploited arable land.

Starch metabolism in potato involves many redundant metabolic routes employing a large number of gene isoforms. Starch biosynthesis takes place both in the leaves and tubers and from previous mRNASeq profiling of potato, it appears that while isoforms of the same enzymes are used in both leaves and tubers, they are largely encoded by two different sets of genes, one set active in the tuber and one in the leaf [1]. Even though the starch biosynthesis has been subject of intense interest for many years and despite extensive studies in both cereals and dicotyledonous plants, it is still not simple to manipulate to obtain higher yields in crop plants. Starch synthesis in potatoes and cereals takes place in specialized plastids, amyloplasts, in storage organs [2]. In the cereal endosperm, it was recently suggested, that the quantitatively important precursor for starch synthesis imported via a specific transporter into the seed endosperm amyloplasts is ADP-glucose, which is subsequently directly incorporated into starch by starch synthase [3]. This route contradicts the classical view of starch synthesis, where the precursor imported into the amyloplast is considered to be hexose phosphates, and is still subjected to some controversy [4] [3]. However, it underscores, that the starch biosynthesis is exhibiting considerably more mechanistic plasticity than previously thought. In potato tubers the precursor for starch synthesis imported into the amyloplast is indeed hexose phosphate. Until recently, transport was believed to take place solely in the form of glucose-6-phosphate (G6P) [5], but convincing evidence for the direct import of glucose-1-phosphate (G1P) into the potato tuber amyloplast was provided by Fettke et al. [6], although the specific transporter protein for G1P is unknown. However, in a comprehensive series of experiments, using the potato cv Desiree as a model, G6P was shown to be the quantitatively important precursor imported into potato tubers under normal growth conditions [6]. Nonetheless, manipulation of the metabolite levels of hexose phosphates by changing expression of the G1P/G6P interconverting enzyme, phophoglucomutase (PGM) in potato tubers, suggests that under conditions that favor high cellular G1P concentrations almost normal starch synthesis can be supported by G1P import [5]–[7].

Basically two different but non-exclusive hypotheses for differential tuber yield can be formulated. Either there is a difference in sucrose load into the tuber from the green parts of the plant, which could be caused by more efficient leaf architecture or differences in photosynthetic efficiency. Or there is a difference in carbon partitioning among starch-, ATP- and protein-production within the potato tuber. In this study we show that differences in yield of different potato cultivars are reflected in specific, consistent and interpretable differences in gene expression in the starch metabolism, most notable plastidial and cytosolic PGM, which can be confirmed by measuring sucrose and hexose phosphate concentrations and the enzymatic PGM activity causing predictable differences in intracellular G1P and G6P levels.

## Methods

### Plant material

Two separate batches of tuber material from three potato cultivars of different yield, Jutlandia (low), Desiree (medium) and Kuras (high) were grown under regular field conditions. The first batch was grown at Try Hedegaard, Try, Denmark. Seed tubers were planted April 22, 2008 and samples were collected during tuberization stage (9 weeks after planting), tuber growth stage (12, 15 and 18 weeks after planting) and tuber maturing stage (21 weeks after planting). To validate the results, a second batch of tubers was analyzed from a different trial. This batch was grown at LKF, Vandel, Denmark in 2009. Seed tubers were planted 23rd of June 2009 and tubers were sampled 9 and 11 weeks after planting. At each time point sampling was performed in biological triplicates (different plants). Tubers were immediately frozen in liquid N_2_ as either whole (small tubers) or quickly following cutting into smaller bits (medium and large tubers). The tissue was stored at −80°C until processing. Following the last sampling, remaining plants were harvested and tuber and yield was determined per hectare.

### RNA extraction

RNA extraction protocol was carried out using the RNAqueous® Kit with a few modifications. 500 µl RNAqueous Lysis buffer was added to 100–200 mg tuber tissue (ground to fine powder in liquid nitrogen) in a Precellys CK14 tissue homogenization tube (including beads) and subjected to 3 cycles of 5 sec (with 5 sec pause in between) homogenization at 6500 rpm using a Precellys mechanical homogenizer (Bertin Technologies, France). Following centrifugation at 12000×g at room temperature for 5 minutes, 400 µl of the supernatant was transferred to a new tube and 500 µl of Phenol∶Chloroform∶Isoamylalcohol (25∶24∶1) was added, mixed and centrifuged for 5 minutes at 12000×g. 200 µl of the top phase was added to 200 µl of 64% ethanol and transferred to an RNAqueous filter. Total RNA was obtained using the RNAqueous® Kit protocol from this step forward. RNA purity was analyzed by 1% TAE-agarose gel electrophoresis and spectroscopic analysis. RNA quality was evaluated by observing the integrity of 28S rRNA and 18S rRNA from the agarose gels. Total RNA concentration was determined by NanoDrop® Spectrophotometer ND-1000 (Thermo Scientific, Wilmington, DE, USA).

### DeepSAGE library construction and sequencing

2 µg of total RNA per sample was used to construct 40 DeepSAGE tag libraries from 2008 and 6 libraries from 2009 [8] using a modification to facilitate direct sequencing of the amplicons by Solexa/Illumina sequencing [9]. The resulting samples were diluted to a final concentration of 10 nM and pooled into four pools each containing 10 samples with a unique identification key for each sample. DNA quantification of pools was performed using the PicoGreen assay (Quant-iT PicoGreen dsDNA Kit, Invitrogen) prior to template DNA hybridization and sequencing on an Illumina Genome Analyzer II (2008 series) or HiSeq2000 (2009 series) (Illumina, San Diego, USA) according to the manufacturer's instructions.

### Data analysis

Sequence files were obtained and converted to sequence tag tables using the perl script SolexaTagExtractionPipeline.pl (all perl scripts are available at www.solanumdata.dk). Following Principal Component Analysis (PCA) of replicates, in order to determine variation and identify possible outliers (none were identified), replicates were pooled into 21 libraries (combined biological replicates, 3 cultivars from 7 different time points summing up to 21 libraries). Expression values were normalized to tags per 2,000,000, since this was close to the smallest library size. The 21 bp sequence tags were annotated by matching to a sequence collection consisting of the newly released potato genome model [1] (DM v 3.4 mRNA transcripts, available at (http://solanaceae.plantbiology.msu.edu/) and genes enclosed in the Solanum Tuberosum Gene Index (StGI release 13.0) using the perl script GlobalSAGEmap-V24.pl.

Using a combination of Kyoto Encyclopedia of Genes and Genomes (KEGG) [10], Plant Metabolic Network (PMN) (www.plantcyc.org) and literature studies, a list of genes involved in starch metabolism together with primary annotation ID (PGSC or STGI) was compiled (see table S1). Gene expression values for these genes were derived from the normalized libraries containing tag tables. In cases where multiple tags were matching a single gene, or multiple isoforms of genes were observed, the tag counts were summed to provide a measure of transcript levels for specific metabolic steps. Further visualization of the expression level was performed by use of Cluster 3.0 and Java TreeView [11].

### Extraction and measurement of sucrose and sugar phosphates

1 ml of cold chloroform∶methanol (3∶7) was added to 500 mg of tuber tissue in Precellys CK14 tubes and subjected to mechanical disruption similarly to the RNA extraction procedure described above. The tubes were weighed before and after the addition of tissue to determine the precise amount of biological material added. The samples were incubated for 2 h at −20°C, before 500 µl ice-cold water was added and the tubes were incubated on ice for 15 minutes with repeated mixing. Following centrifugation at 12000×g at 4°C for 20 minutes, 1200 µl of the upper phase was transferred to a new tube. The lower chloroform phase was reextracted with 400 µl ice cold water and the upper phase was combined with the first amount in a new tube. Finally, the samples were evaporated to dryness in a vacuum drier and reconstituted in 100 µl water.

The samples and standards were analyzed in six replicates of sample preparation from the same plant on a Dionex DX500 HPLC system (Sunnyvale, CA, USA) using a Carbopac PA10 carbohydrate column (Dionex, Sunnyvale, CA, USA) and electrochemical detection. The mobile phase consisted of 50 mM sodium hydroxide, 300 mM sodium acetate and elution was conducted at a flow rate of 0.25 ml/min [12]. Peaks of interest were integrated using PeakNet (Sunnyvale, CA, USA) and converted to concentrations using standard curves established on the same system from analytical grade reference standards obtained from Sigma-Aldrich (St.Louis, MO, USA).

### PGM enzyme assay

Tuber samples (the same as for RNA extraction above) were taken from all three cultivars from 5 different time points: 12, 15 and 18 weeks after planting (2008 series) and 9 and 11 weeks after planting (2009 series). Small pieces of tubers were placed in a pre-cooled mortar and ground to fine powder in liquid nitrogen. 200 mg of homogenized tissue was subsequently transferred into a 2 ml microcentrifuge tube and mixed with 200 µl of extraction buffer (20 mM Tris-HCl pH 8.0, 4 mM DTT, 5 mM MgCl_2_) followed by centrifugation at 14000 rpm for 4 minutes. The supernatant was transferred to a new tube followed by another centrifugation step. The resulting supernatant was diluted 5× and 10× with extraction buffer and 8 µl was added to a flat-bottomed 96 well UV plate. 200 µl of 20 mM Tris-HCl pH 7.5, 10 mM MgCl_2_, 1 mM K_2_-G1P, 0.25 mM NADP^+^, 0.1 mM G1,6P_2_ and 1 unit of G6P-dehydrogenase was added. The plate was inserted into a Tecan Infinite M1000 (Tecan, Mannedorf, Switzerland) spectrofotometer. Absorbance was measured at 340 nm. Negative controls without sample were analyzed in parallel and reaction rates were calculated by subtracting the control sample value from the assay sample values.

### Statistical analysis

Principal Component Analysis (PCA) of replicates was carried out using The Unscrambler® X ver 10.1 (CAMO Software, Oslo, Norway). The student t-tests were performed according to the algorithm provided by Microsoft Excel (Microsoft Corporation, Seattle, WA, USA). The term significant is only used for p-values<0.05.

## Results

### Field grown potato cultivars

Tuber yield per hectare was calculated by scaling up the average yield of ten plants at the end of the growth season of 2008 (see table 1). Starch is the main dry matter component of potato tuber ranging from 10–26% (w/w) within most varieties of commercial use [13]. In relation to, the low yielding cv Jutlandia (380 hkg/ha, 11.7% starch), the medium yielding cv Desiree has 66% higher starch yield (478 hkg/ha, 15.4% starch) and high yielding cv Kuras has up to 180% yield increase (596 hkg/ha, 20.9% starch) compared to cv Jutlandia. It should be noted that the time of planting as well as natural variation in growing conditions including weather and soil conditions exist between the 2008 and 2009 field trial series. Therefore, the time points of sampling are not directly comparable. In fact, the weather in Denmark during the growth period up to sampling was more favorable in 2009 and thus the tubers were likely to be slightly more mature at the sampled time points compared to 2008.

**Table 1:** Sample statistics.

cultivar	time point	replicate 1	replicate 2	replicate 3	combined	tuber yield (hkg/ha)	starch content (%)
**Desiree (2008)**	**9**	826,191	1,661,343	973,030	3,460,564		
**Desiree (2008)**	**12**	411,461	NA	892,609	1,304,070		
**Desiree (2008)**	**15**	278,536	885,199	904,277	2,068,012	380	11.9
**Desiree (2008)**	**18**	1,049,772	1,277,006	NA	2,326,778		
**Desiree (2008)**	**21**	586,672	983,331	1,052,849	2,622,852		
**Desiree (2009)**	**9**	7,644,714	11,630,251	9,769,669	29,044,634		
**Desiree (2009)**	**11**	16,693,106	12,714,866	10,440,036	39,848,008		
**Jutlandia (2008)**	**9**	NA	533,883	1,433,784	1,967,667		
**Jutlandia (2008)**	**12**	1,133,434	852,047	1,044,402	3,029,883		
**Jutlandia (2008)**	**15**	1,050,297	761,643	698,728	2,510,668	478	15.4
**Jutlandia (2008)**	**18**	578,588	1,095,558	1,291,794	2,965,940		
**Jutlandia (2008)**	**21**	NA	607,322	838,688	1,446,010		
**Jutlandia (2009)**	**9**	15,674,527	13,134,880	9,724,414	38,533,821		
**Jutlandia (2009)**	**11**	17,166,126	10,144,737	12,100,324	39,411,187		
**Kuras (2008)**	**9**	1,109,293	1,988,857	2,368,777	5,466,927		
**Kuras (2008)**	**12**	970,045	1,034,725	513,293	2,518,063		
**Kuras (2008)**	**15**	796,137	213,351	NA	1,009,488	596	20.9
**Kuras (2008)**	**18**	457,545	1,116,289	921,391	2,495,225		
**Kuras (2008)**	**21**	393,784	463,443	287,787	1,145,014		
**Kuras (2009)**	**9**	12,593,044	12,071,757	12,555,532	37,220,333		
**Kuras (2009)**	**11**	10,970,940	67,110,212	8,743,707	86,824,859		

Total number of DeepSAGE tags determined for each library for Desiree, Jutlandia and Kuras cultivars. Data are represented for all three biological replicates for each time point: 9, 12, 15, 18 and 21 weeks after planting (2008) and 9 and 11 weeks after planting (2009). Combined tag counts of biological replicates are also presented. The last columns represent the tuber yield in hkg/ha and starch content of Jutlandia, Desiree and Kuras cultivars.

### Sampling and data generation

Of the 51 samples planned, 46 yielded sequence data in the end. At one sample time point, no tubers were available for sampling, in other four other cases, RNA of acceptable quality was not obtained. The RNA quality was evaluated by gel electrophoresis and spectrophotometric measurements. In general, the yield of RNA decreases about 2.5 fold with tuber age (see figure S1). Samples were approved if acceptable bands of 28S and 18S rRNA were observed in gel electrophoresis (see figure S2A). All 46 RNA samples yielded a DeepSAGE tag library which was subjected to sequencing. Following bioinformatic sorting according to nucleotide identification keys of the obtained sequence reads, an average of 908,429 tags were obtained per sample varying from 213,351 to 2,368,777 for the 2008 series (Genome Analyzer II) and 7,644,714 to 67,110,212 (HiSeq2000) see Table 1). The approximately 10 fold difference in sequencing depth obtained between the two sequencing platforms is simply reflecting the increased throughput of the HiSeq2000 compared to the Genome Analyzer II. The rather high variation of tags obtained within each sequencing platform was a result of imperfect stoichiometric pooling of samples prior to sequencing. When biological replicates were summed, the variance of tags obtained per sample time point decreased two to three fold with values between 1,009,488 and 5,466,927; and 29,044,634 and 86,824,859, respectively (see Table 1). Of these libraries between 0.59% and 2.48% of tags obtained were derived from genes involved in starch metabolism (see table S2) and constitute the focus of this study (see table S3 for tag counts summed to genes and table S2 for raw tag counts).

### Gene expression of starch metabolism enzymes

**Figure 1:**
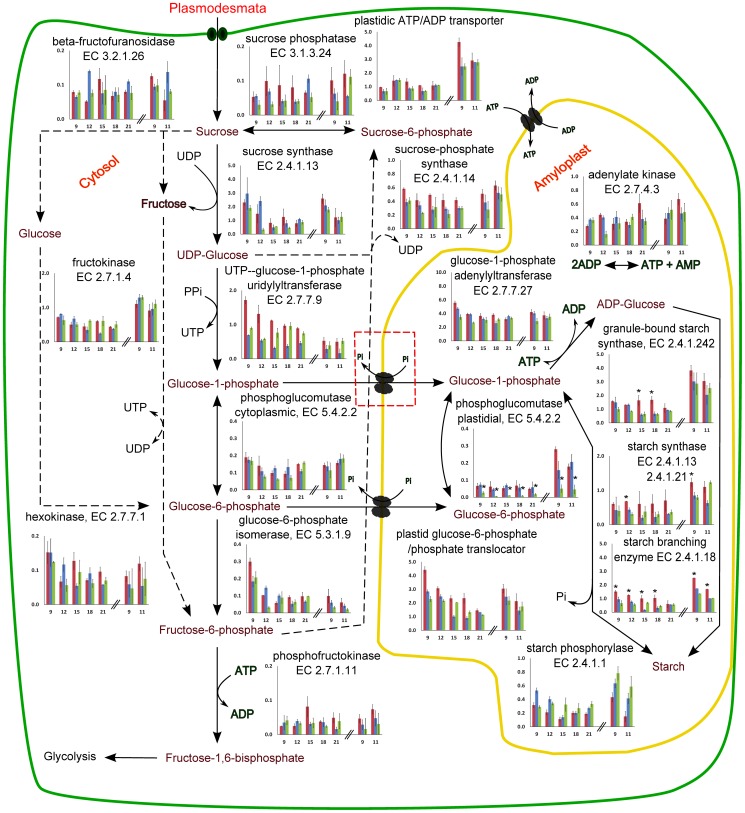
Potato tuber gene expression in starch metabolism throughout tuber bulking. SAGE tags were mapped to genes encoding enzymes involved in starch metabolism. Tag counts per million (y-axis) for multiple enzyme isoforms catalyzing a single step were summed. Desiree is shown in red, Jutlandia in blue and Kuras in green. The error bars indicate standard error of measurement. Numbers on x-axis indicate weeks after planting. Left side of the hatched bars: 2008; right side: 2009. The asterisk symbol indicates gene expression values significantly different than the gene expression of the two remaining cultivars (p-value<0.05). The solid reaction arrows indicate the major metabolic route of high carbon flux leading to starch accumulation. The hatched lines indicate minor metabolic routes where carbon flux is limited. The red hatched box indicates the G1P transporter suggested by Fettke and coworkers and discussed in this study. The green solid line indicate cell membrane, the yellow line indicates the amyloplastic membrane.

Figure 1 shows a metabolic map of the starch metabolism overlaid with the normalized gene expression values. Overall, a rather little change in gene expression over time and between cultivars was observed with few notable exceptions. First, sucrose synthase (EC 2.4.1.13) and plastidial G6P/P translocator had 1.5–4 fold higher expression in early (9 and 12 weeks) than in later time points (15, 18 and 21 weeks). Second, expression of G6P-isomerase (EC 5.3.1.9) was 2–8 fold higher at early time points compared to late.

The most remarkable difference between cultivars, however, was found in the amyloplast. Whereas most plastidial genes had a rather constant expression among the cultivars and time points, plastidial PGM was found in very low amounts in Kuras compared to the two lower yielding cultivars (up to 10 fold difference between Desiree and Kuras at Week 12, 2008 series, p-value = 3,7×10^−6^). Additionally, in Desiree the expression of starch synthase (EC 2.4.1.242, 2.4.1.13, 2.4.1.21) and starch branching enzyme (EC 2.4.1.18) were found to be 2–3 fold higher at most time points (see Table S3 and Figure 1) in the 2008 series. Importantly, the robustness of these gene expression measurements was confirmed by the 2009 series, which was found to be in remarkable agreement with the 2008 series. Noteworthy, however, is a general upregulation of the plastidial starch synthetic route (granule bound starch synthase, starch synthase and starch branching enzyme) as well as starch phosphorylase in 2009, possibly reflecting the more favorable growth conditions.

### Enzyme assay

**Figure 2:**
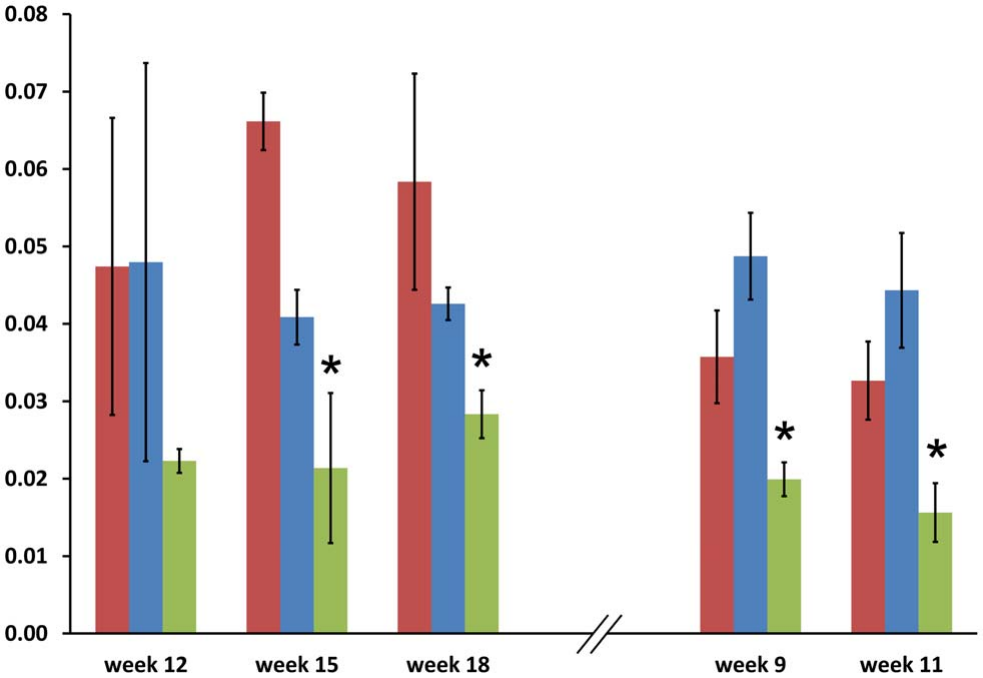
Total phosphoglucomutase activity. The bar chart represents the differences between potato cultivars in respect to total phosphoglucomutase activity (the unit is A340/min). Left side of the hatched bars: 2008; right side: 2009. Significant differences (p-value<0.05) between Kuras and two other cultivars are indicated with asterisks. Desiree is shown in red, Jutlandia in blue and Kuras in green. The error bars indicate standard error of measurement.

Purification of intact plastids from potato tubers is only possible for very young tubers or *in vitro* grown tubers, hence specific determination of plastidial PGM (pPGM)is not feasible. Therefore to verify that differences in PGM transcript abundance were indeed also reflected in differences at the protein level, total phosphoglucomutase (tPGM) activity (including both plastidial and cytosolic) was determined for all three cultivars at time points 12, 15 and 18 weeks after planting (2008) and for 9 and 11 weeks after planting (2009). Consistent with the gene expression data, Kuras had an average 2.17 fold lower (p-value = 0.00061) tPGM activity than the other two cultivars in 2008 and 2.28 fold lower (p-value = 0.00034) in 2009 (see Figure 2). This enzyme activity was in excellent agreement with the combined gene expression level for both cytosolic and plastidial phosphoglucomutase at 12, 15 and 18 weeks after planting, which for Kuras was on average 2.25 fold (2008) and 1.83 fold (2009) lower than for other two cultivars.

### Sucrose and hexose phosphates profiling

**Figure 3:**
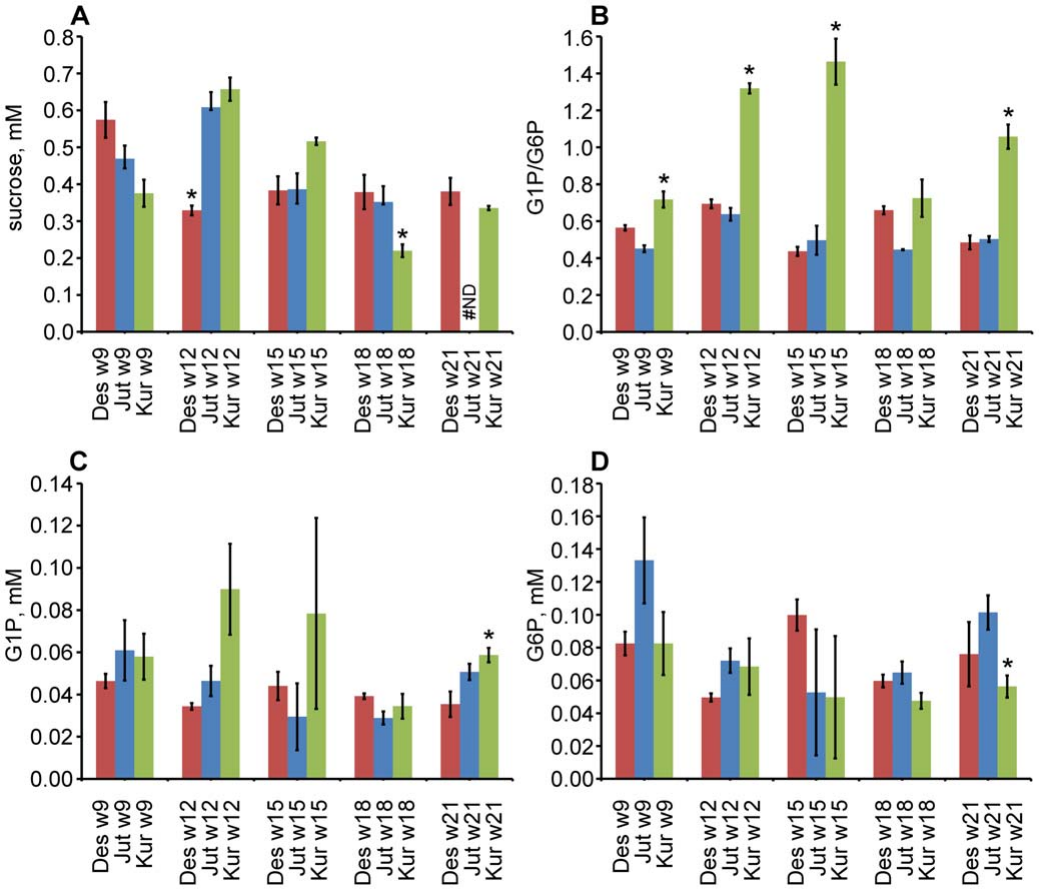
Steady state tuber concentration of sucrose and glucose phosphates. A. Sucrose concentrations A: Example chromatogram indicating G1P and G6P peaks. B: G1P/G6P ratios. C: G1P concentrations. D: G6P concentrations. Error bars indicate standard error of measurement. The asterisk indicates metabolite levels significantly different for Kuras versus Desiree and Jutlandia (p-value<0.05). The determination of G1P/G6P is much more accurate than individual determination of G1P and G6P (see text for detail).

To determine whether the difference in plastidial PGM activity lead to higher or lower steady state concentrations of G1P methanol/chloroform extraction of soluble molecules from tuber samples was performed and the concentrations of G1P and G6P were determined by HPLC. From Figure 3B it can be seen that Kuras during the tuber bulking period at 12, 15 and 21 weeks had a 2–2.5 fold higher G1P to G6P ratio than Jutlandia and Desiree. By determining both the G1P and G6P concentrations simultaneously in the same chromatogram, thereby excluding random variations in sample preparation and loading, the ratio of G1P and G6P was much more reliably determined than the individual concentrations and thus reached statistical significance. Therefore, the individual concentrations of G1P (Figure 3C) and G6P (Figure 3D) displayed a much higher variation than the corresponding ratios between them and the differences are not statistically significant. Nonetheless, it can be seen that the difference in ratio of G1P and G6P does not seem to be caused by lowering of the G6P concentration in Kuras, but in fact due to a higher G1P concentration for week 12, 15 and 21. Somewhat puzzling Kuras did not show elevated G1P/G6P ratio at week 18 as should be expected from the gene expression and enzyme activity assays, but at this time point the sucrose concentration (Figure 3A) was also lower than observed for other time points. This suggests that for unknown reasons, possibly related to specific growth conditions, the sucrose flow into the tubers may have decreased at this specific sample point, thus influencing the G1P and G6P levels downstream of the biochemical pathway. Such low sucrose levels was also observed for Desiree week 12, and results in slightly loweved G1P and G6P concentrations at this time point as well.

## Discussion

The purpose of this study was to elucidate, whether differential yield of potato cultivars was reflected in differences in gene expression in the starch biosynthesis in tubers. To elucidate this we profiled the gene expression of genes involved in starch metabolism in tubers of a high (Kuras), a medium (Desiree) and a low (Jutlandia) yielding potato cultivar. These three cultivars are not closely related compared to other elite potato cultivars (http://www.plantbreeding.wur.nl) (Kuras ID 3847; Desiree ID 2025; Jutlandia ID 3464)). Decreasing yield of RNA was obtained with tuber age (see figure S1). This is most likely reflecting that as bulking is progressing, more and more of the tuber sample is constituted of starch and hence, decreasing amounts of the mRNA contained in the cytoplasm is sampled. Following tag extraction, and annotation, tags matching starch genes were extracted and they constituted between 0.59% and 2.48% of all tags. Tags matching isoforms of genes were summed to represent a specific metabolic step. This variance in starch transcripts was not correlated with tuber age or number of tags sequenced. However noteworthy is the fact, that the highest yielding cv Kuras had the lowest average fraction of starch synthesis related tags (mean = 1.01%±0.01%) followed by the low yielding Jutlandia (1.09%±0.02%) and the medium yielding Desiree (1.35%±0.02%) (2008 series). Therefore, the overall fraction of transcripts involved in starch synthesis and hence the overall gene expression level of the metabolic pathway was not generally positively correlated with yield.

Investigating the expression of individual genes revealed that most of them are not significantly different between the three cultivars (Figure 1 and Table S3). Interestingly however, two enzymes involved in the final steps of starch synthesis, starch synthase and starch branching enzyme were expressed at a higher level in the medium yielding cv Desiree, compared to the other two cultivars (at least in the 2008 series). This provides an explanation to why Desiree is producing more starch than Jutlandia: The high expression of these enzymes causes a higher turnover of G1P increasing the sink strength of the plastid. This does not, however, explain the highest yield of Kuras, since the expression level of these enzymes in this cultivar was similar to the lowest yielding cultivar. The most striking difference between Kuras and the other cultivars is a very low expression pPGM during the tuber growth period resulting in a low tPGM enzyme activity at these time points. At first this seems difficult to reconcile with the high starch yield, since a low level of pPGM constitutes a bottleneck in the classical view of hexose phosphate import (as G6P) into the amyloplast [14]. However, recently it was shown that plastidial hexose phosphate import can occur in the form of G1P and that exogenously supplied G1P is incorporated into starch faster than G6P [6]. The transporter responsible for G1P is presently not known. Interestingly, in Desiree, silencing of either plastidial [14] or cytoplasmic [5] PGM results in a low starch phenotype, whereas the simultaneous silencing of both results in normal levels of starch [7]. These results can be explained if direct import of G1P is considered (see figure 1): silencing of pPGM results in a bottleneck in plastidial G1P synthesis and hence a low concentration of starch precursors. Transcriptional silencing of cytoplasmic PGM results in decreased cytoplasmic G6P concentration and hence poor import of G6P into the plastid via the G6P transporter. G1P may be imported directly, but in the presence of high amounts of pPGM there is a competing reaction between starch synthesis and conversion to G6P. The ΔG° for the conversion of G1P to G6P is −7.28 kJ/mol thus shifts the G1P/G6P ratio towards G6P. Silencing both plastidial and cytoplasmic PGM results in elevated levels of intracellular G1P and cause increased import into the plastid via the G1P transporter. Once inside the plastid, there is little conversion to G6P and thus a higher concentration of starch precursors is present to support starch synthesis. Therefore, a possible explanation to how Kuras can obtain high rates of starch synthesis with low levels of pPGM (and normal levels of cPGM) could therefore depend on the levels of G1P in Kuras. Indeed, by measuring the concentrations of cellular G1P and G6P we found that Kuras contains an elevated ratio of G1P/G6P compared to Jutlandia and Desiree at week 12, 15 and 21. Therefore, the results support the hypothesis, that in contrast to Desiree, import of G1P is quantitatively important in Kuras. A higher plastidial G1P concentration is likely to cause an increased flux of carbon through the classical starch synthesis route involving glucose pyrophosphorylase (AGPase) and starch synthase if ATP is available. Indeed it was found that in Desiree that the ATP/ADP translocator displays the greatest control over starch synthesis and altered adenylate pools results in altered starch synthesis [15]–[17]. It is intriguing however, that Fettke et al. showed evidence for the existence of an ATP independent starch synthesis route by incorporating G1P directly into starch via starch phosphorylase [6]. Generally, starch phosphorylase is considered to be a starch degrading enzyme, but if elevated levels of G1P are present, as in Kuras, the synthesis reaction may likely be favored. In this study, we found that Kuras (and Jutlandia) in contrast to Desiree under the more favorable conditions of 2009 upregulated the starch phoshorylase as well as the starch synthase and starch branching enzyme expression. Taken together our results indicate the possibility, that a quantitatively significant part of the starch synthesized by Kuras, in contrast to Desiree, is partitioned via the ATP-independent pathway. However, more studies are needed to elucidate this.

## Conclusion

Overall we found that specific differences in gene expression levels between potato cultivars exist. This raises concerns as to what extent the extensive studies performed using only Desiree as a model is valid for all other potato variants. Indeed, early contradictory evidence obtained by Stark et al. [18] and Sweetlove et al. [19] on the importance of AGPase also suggests that different cultivars may have different rate limiting steps for starch synthesis. In the case of Kuras, we found support for quantitative importance of G1P import into the plastid. The gene(s) responsible for the translocation of G1P is still unknown and consequently has not been the focus of any studies in potato, but may indeed constitute a highly interesting target in some cultivars. Indeed, a truly interesting perspective is that the potential ATP independent starch synthesis pathway may have quantitative importance in some high yielding cultivars, including Kuras. This opens entirely new possibilities for metabolic engineering of the starch metabolism in potato, potentially leading to increased yields and space efficiency of this crop.

## Supporting Information

Figure S1 **Total amount of extracted RNA throughout tuber bulking versus time point**. Total RNA yield (average from biological replicates, n = 3) during tuber development as determined by spectophotometry. Red indicates Desiree, blue Jutlandia and green Kuras.
(TIFF)

Figure S2 **TAE-Agarose gel pictures.** A: Two representative examples of RNA preparations from potato tuber. B: Two representative examples of DeepSAGE library tag generations: productive linkerA-tag-linkerB is 96 nt, spurious dilinker is 73 nt.
(TIFF)

Table S1 **List of gene IDs and gene names relevant for starch metabolism.**
(DOC)

Table S2 **All tags and tag counts involved in starch metabolism.**
(DOCX)

Table S3 **Summed tag counts for multiple enzyme isoforms catalyzing a single step in starch metabolism.** Table outlines the essential genes in starch metabolism. Primary annotation names marked with asterisk indicate that different tags matching multiple enzyme isoforms were taken together and their counts were summed. Tag counts represent libraries of combined biological replicates.
(XLS)
