# Supplementary figures and images for: Transcriptome Analysis Suggests That Starch Synthesis May Proceed via Multiple Metabolic Routes in High Yielding Potato Cultivars
Source: PLoS One. 2012 Dec 17;7(12):e51248. doi: 10.1371/journal.pone.0051248 (PMC3524171; doi:10.1371/journal.pone.0051248)

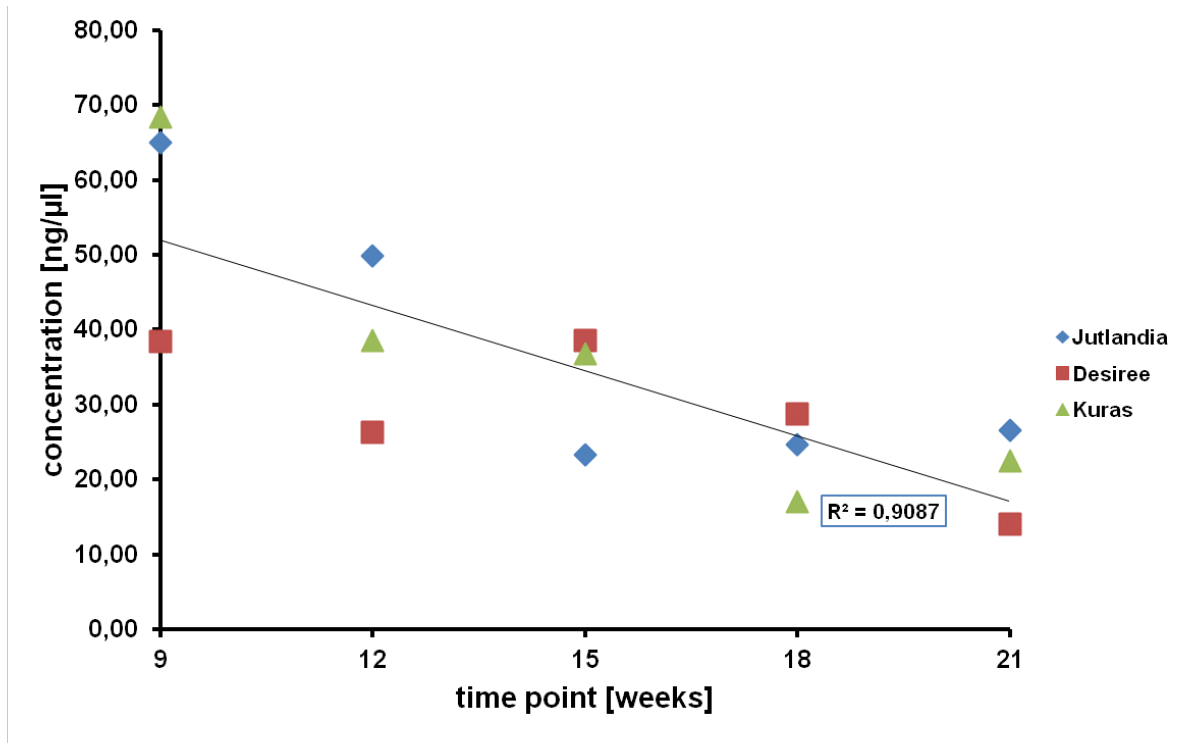

Supplement: Figure S1 — Total amount of extracted RNA throughout tuber bulking versus time point. Total RNA yield (average from biological replicates, n = 3) during tuber development as determined by spectophotometry. Red indicates Desiree, blue Jutlandia and green Kuras. (TIFF) [file pone.0051248.s001.tif]

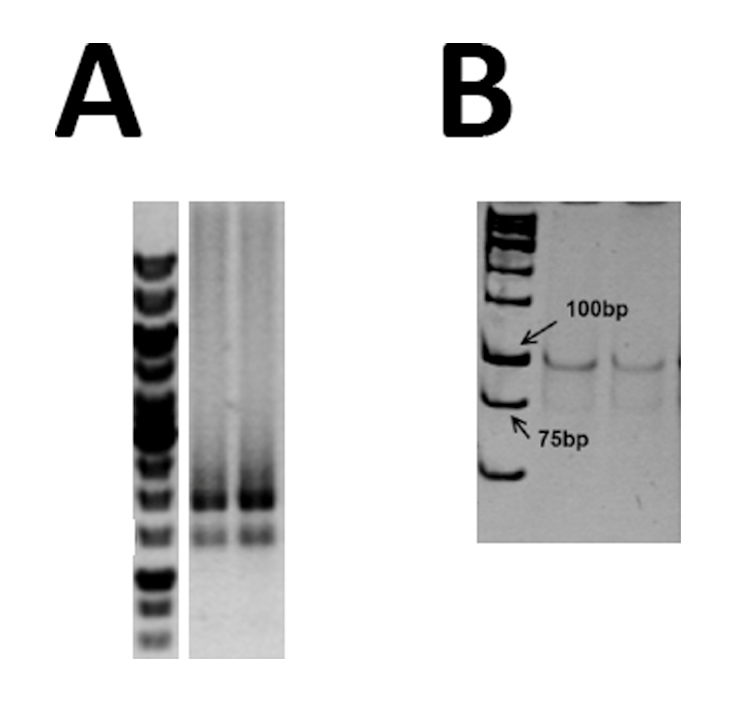

Supplement: Figure S2 — TAE-Agarose gel pictures. A: Two representative examples of RNA preparations from potato tuber. B: Two representative examples of DeepSAGE library tag generations: productive linkerA-tag-linkerB is 96 nt, spurious dilinker is 73 nt. (TIFF) [file pone.0051248.s002.tif]
