# Supplementary material for: Transcriptome Analysis Suggests That Starch Synthesis May Proceed via Multiple Metabolic Routes in High Yielding Potato Cultivars
Source: PLoS One. 2012 Dec 17;7(12):e51248. doi: 10.1371/journal.pone.0051248 (PMC3524171; doi:10.1371/journal.pone.0051248)
Supplement: Table S1 — List of gene IDs and gene names relevant for starch metabolism. (DOC) [file pone.0051248.s003.doc]

**Supporting table 1**

| **Primary Annotation ID** | **Primary Annotation Name** |
| --- | --- |
| PGSC0003DMG400009981 | 1,4-alpha-glucan branching enzyme |
| PGSC0003DMG400010664 | 1,4-alpha-glucan-maltohydrolase |
| PGSC0003DMG400016589 | 4-alpha-glucanotransferase, chloroplastic/amyloplastic |
| TC196558,TC223475 | 4-alpha-glucanotransferase, homologue to UniRef100_Q6R608 Cluster |
| PGSC0003DMG400025455 | 6-phosphofructokinase 1 |
| PGSC0003DMG400016208  PGSC0003DMG402023631 | 6-phosphofructokinase 5, chloroplastic |
| PGSC0003DMG400026110 | Adenylate kinase |
| PGSC0003DMG400028992 | Adenylate kinase 1 chloroplast |
| PGSC0003DMG400013933 | Adenylate kinase 2, chloroplastic |
| PGSC0003DMG400001247  PGSC0003DMG400015187 | Adenylate kinase B |
| PGSC0003DMG400013379  PGSC0003DMG400020217 | Adenylate kinase family |
| PGSC0003DMG400007496  PGSC0003DMG400027404 | Adenylate kinase isoenzyme 6 |
| PGSC0003DMG400027906 | Adenylate kinase, chloroplastic |
| PGSC0003DMG400007974  PGSC0003DMG400009891  PGSC0003DMG400020603  PGSC0003DMG401017626 | Alpha-amylase |
| GH638277,TC197475 | Alpha-glucan phosphorylase, H isozyme, UniRef100_P32811 Cluster |
| PGSC0003DMG400000169  PGSC0003DMG400001549  PGSC0003DMG400024145  PGSC0003DMG400026199 | Beta-amylase |
| PGSC0003DMG400001855  PGSC0003DMG400012129  PGSC0003DMG402020509 | Beta-amylase PCT-BMYI |
| PGSC0003DMG400009936  PGSC0003DMG402028252  PGSC0003DMG400002756  PGSC0003DMG400019494 | Beta-fructofuranosidase |
| PGSC0003DMG400033142 | Beta-fructofuranosidase, cell wall isozyme |
| PGSC0003DMG400033142 | Beta-fructofuranosidase, insoluble isoenzyme CWINV3 |
| PGSC0003DMG400020361  PGSC0003DMG400024246  PGSC0003DMG400026916  PGSC0003DMG400028311 | Fructokinase |
| PGSC0003DMG400027017 | Fructokinase 2 |
| PGSC0003DMG400030653 | Fructokinase 3 |
| PGSC0003DMG400000735  PGSC0003DMG400009026 | Glucose-1-phosphate adenylyltransferase |
| PGSC0003DMG400015952 | Glucose-1-phosphate adenylyltransferase large subunit 2, chloroplastic/amyloplastic |
| PGSC0003DMG400031084 | Glucose-1-phosphate adenylyltransferase small subunit, chloroplastic/amyloplastic |
| BI919490 | Glucose-1-phosphate adenylyltransferase, UniRef100_O04924 Cluster |
| PGSC0003DMG400002750  PGSC0003DMG400010802 | Glucose-6-phosphate 1-dehydrogenase |
| PGSC0003DMG400017394 | Glucose-6-phosphate 1-dehydrogenase, chloroplastic |
| PGSC0003DMG400020269 | Glucose-6-phosphate 1-dehydrogenase, cytoplasmic isoform |
| PGSC0003DMG400012910 | Glucose-6-phosphate isomerase |
| CV429428 | Glucose-6-phosphate isomerase, homologue to UniRef100_Q68HC9 Cluster |
| PGSC0003DMG400001041  PGSC0003DMG400005602  PGSC0003DMG400044320  PGSC0003DMG402012710  PGSC0003DMG400001041 | Glucose-6-phosphate/phosphate translocator 1, chloroplast |
| PGSC0003DMG400005269 | Glucose-6-phosphate/phosphate translocator 2 |
| PGSC0003DMG400012111 | Granule-bound starch synthase 1, chloroplastic/amyloplastic |
| PGSC0003DMG400001328 | Granule-bound starch synthase 2, chloroplastic/amyloplastic |
| PGSC0003DMG400016521  PGSC0003DMG400030624 | Hexokinase |
| PGSC0003DMG400016521 | Hexokinase 2 |
| PGSC0003DMG400000295 | Hexokinase 5 |
| PGSC0003DMG400013187 | Hexokinase 6 |
| PGSC0003DMG400002525 | Hexokinase 7 |
| PGSC0003DMG400003103  PGSC0003DMG400003514  PGSC0003DMG400007913  PGSC0003DMG400014208  PGSC0003DMG400026784  PGSC0003DMG400030682  PGSC0003DMG401028529 | Inorganic pyrophosphatase |
| PGSC0003DMG400008932 | Magnesium dependent soluble inorganic pyrophosphatase |
| PGSC0003DMG400004999  PGSC0003DMG400012223 | Soluble inorganic pyrophosphatase |
| PGSC0003DMG400008943 | Invertase |
| PGSC0003DMG400020699 | Isoamylase isoform 1 |
| PGSC0003DMG400000954 | Isoamylase isoform 2 |
| PGSC0003DMG401007274  PGSC0003DMG402007274 | Isoamylase isoform 3 |
| CN212714 | Isoamylase isoform 3, homologue to UniRef100_Q84YG5 Cluster |
| PGSC0003DMG400017617  PGSC0003DMG400026781 | Isoamylase N-terminal domain containing protein |
| PGSC0003DMG400016208  PGSC0003DMG400017413  PGSC0003DMG400019734  PGSC0003DMG400025455 | Phosphofructokinase |
| PGSC0003DMG401000669 | Pyrophosphate-dependent phosphofructokinase beta subunit |
| PGSC0003DMG400001912  PGSC0003DMG400024224 | Phosphoglucomutase |
| TC196442 | Phosphoglucomutase, chloroplast, UniRef100_Q9M4G5 Cluster |
| TC202272 | Phosphoglucomutase, cytoplasmic, UniRef100_Q9M4G4 Cluster |
| PGSC0003DMG400028641  PGSC0003DMG400005612 | Plastidic ATP/ADP-transporter |
| PGSC0003DMG400009861 | Plastidic hexokinase |
| PGSC0003DMG402018552 | Soluble starch synthase 1, chloroplastic/amyloplastic |
| CK717167 | Soluble starch synthase 3, chloroplast, UniRef100_Q43846 Cluster |
| PGSC0003DMG400016481 | Soluble starch synthase 3, chloroplastic/amyloplastic |
| PGSC0003DMG400022307 | Starch branching enzyme |
| NP005518 | starch branching enzyme II GB|AJ011885.1|CAB40743.1 |
| TC194619 | Starch branching enzyme II, UniRef100_Q9XGA6 Cluster |
| TC194616 | Starch branching enzyme II, UniRef100_Q9XGA8 Cluster |
| PGSC0003DMG400008322 | Starch synthase IV |
| PGSC0003DMG400030619 | Starch synthase V |
| PGSC0003DMG401013540  PGSC0003DMG402013540 | Starch synthase VI |
| PGSC0003DMG400002895  PGSC0003DMG400006672  PGSC0003DMG400016730  PGSC0003DMG400031046 | Sucrose synthase |
| PGSC0003DMG400013546 | Sucrose synthase 2 |
| PGSC0003DMG400021341  PGSC0003DMG400028134 | Sucrose-phosphatase |
| PGSC0003DMG400021341 | Sucrose-phosphatase 1 |
| PGSC0003DMG400026428 | Sucrose-phosphate synthase isoform B |
| PGSC0003DMG402019060 | Sucrose-phosphate synthase isoform C |
| CX162034 | Sucrose-phosphate synthase isoform C, UniRef100_Q3HLN3 Cluster |
| PGSC0003DMG400027936 | Sucrose-phosphate-synthase |
| PGSC0003DMG400001448 | UDP-glucose dehydrogenase 2 |
