# Supplementary material for: Transcriptome Analysis Suggests That Starch Synthesis May Proceed via Multiple Metabolic Routes in High Yielding Potato Cultivars
Source: PLoS One. 2012 Dec 17;7(12):e51248. doi: 10.1371/journal.pone.0051248 (PMC3524171; doi:10.1371/journal.pone.0051248)
Supplement: Table S2 — All tags and tag counts involved in starch metabolism. (DOCX) [file pone.0051248.s004.docx]

| **Year** | **2008** | | | | | | | | | | | | | | | **2009** | | | | | |
| --- | --- | --- | --- | --- | --- | --- | --- | --- | --- | --- | --- | --- | --- | --- | --- | --- | --- | --- | --- | --- | --- |
| **Primary Annotation Name** | **Des w9** | **Des w 12** | **Des w15** | **Des w18** | **Des w21** | **Jut w9** | **Jut w12** | **Jut w15** | **Jut w18** | **Jut w21** | **Kur w9** | **Kur w12** | **Kur w15** | **Kur w18** | **Kur w21** | **Des w9** | **Des w11** | **Jut w9** | **Jut w11** | **Kur w9** | **Kur w11** |
| 1,4-alpha-glucan-maltohydrolase | 60 | 60 | 167 | 203 | 277 | 63 | 123 | 61 | 280 | 320 | 151 | 461 | 321 | 87 | 115 | 2711 | 3241 | 1795 | 2889 | 2338 | 2499 |
| 4-alpha-glucanotransferase, chloroplastic/amyloplastic | 1325 | 1004 | 758 | 1088 | 781 | 1502 | 1279 | 827 | 840 | 1051 | 725 | 520 | 573 | 688 | 661 | 22891 | 15867 | 19528 | 26576 | 15992 | 17808 |
| 4-alpha-glucanotransferase, UniRef100_Q6R608 Cluster | 17 | 25 | 23 | 28 | 44 | 91 | 101 | 117 | 105 | 159 | 44 | 43 | 129 | 48 | 62 | 316 | 28 | 1119 | 1943 | 898 | 1405 |
| 6-phosphofructokinase 5, chloroplastic | 9 | 8 | 18 | 9 | 40 | 33 | 6 | 29 | 10 | 4 | 16 | 17 | 8 | 11 | 16 | 473 | 168 | 235 | 110 | 369 | 629 |
| Adenylate kinase* | 554 | 884 | 621 | 682 | 1224 | 745 | 806 | 821 | 586 | 760 | 728 | 319 | 637 | 821 | 696 | 473 | 110 | 235 | 369 | 168 | 629 |
| Alpha-amylase | 325 | 834 | 596 | 365 | 468 | 374 | 385 | 460 | 1239 | 798 | 231 | 1556 | 861 | 486 | 844 | 13016 | 3583 | 13189 | 6428 | 9623 | 5957 |
| Alpha-glucan phosphorylase* | 631 | 420 | 223 | 405 | 375 | 1058 | 803 | 278 | 402 | 542 | 583 | 681 | 648 | 543 | 666 | 12948 | 4508 | 18993 | 12429 | 23435 | 17576 |
| Beta-amylase | 185 | 253 | 152 | 133 | 112 | 120 | 128 | 76 | 114 | 29 | 189 | 283 | 221 | 135 | 105 | 4665 | 2105 | 2524 | 1304 | 3006 | 2711 |
| Beta-amylase PCT-BMYI | 28 | 77 | 36 | 25 | 50 | 13 | 24 | 22 | 79 | 13 | 46 | 71 | 26 | 51 | 29 | 123 | 470 | 444 | 138 | 311 | 625 |
| Beta-fructofuranosidase* | 157 | 103 | 234 | 135 | 159 | 129 | 280 | 151 | 159 | 219 | 155 | 152 | 170 | 142 | 153 | 3753 | 1644 | 2832 | 4123 | 2946 | 2415 |
| Fructokinase* | 1434 | 1005 | 896 | 1202 | 878 | 1624 | 1350 | 695 | 490 | 760 | 1263 | 1010 | 1232 | 1224 | 1020 | 33211 | 27432 | 38738 | 28802 | 38971 | 33283 |
| Glucose-1-phosphate adenylyltransferase* | 11137 | 7842 | 7287 | 7609 | 6331 | 9411 | 7784 | 6422 | 5118 | 7177 | 6963 | 5350 | 6108 | 6286 | 6666 | 126112 | 111678 | 120358 | 98963 | 86428 | 105842 |
| Glucose-6-phosphate 1-dehydrogenase* | 714 | 1556 | 1662 | 1646 | 1566 | 990 | 1760 | 1512 | 1318 | 1606 | 1036 | 2014 | 1760 | 1734 | 1668 | 18812 | 13231 | 14198 | 15669 | 10740 | 16693 |
| Glucose-6-phosphate isomerase | 598 | 206 | 116 | 184 | 194 | 368 | 294 | 202 | 106 | 132 | 414 | 64 | 178 | 128 | 194 | 2959 | 1840 | 1787 | 1243 | 975 | 607 |
| Glucose-6-phosphate/phosphate translocator* | 8840 | 6134 | 4662 | 4696 | 2906 | 5678 | 4944 | 2010 | 1786 | 2620 | 4558 | 4318 | 4074 | 2648 | 2242 | 91263 | 63891 | 73514 | 43198 | 64949 | 52110 |
| Granule-bound starch synthase, chloroplastic/amyloplastic* | 3147 | 2637 | 3278 | 3355 | 2182 | 2996 | 2645 | 1200 | 1318 | 1869 | 1979 | 1693 | 1292 | 1271 | 1726 | 114585 | 91409 | 90770 | 61334 | 85935 | 76182 |
| Hexokinase* | 304 | 134 | 255 | 142 | 192 | 303 | 234 | 109 | 182 | 116 | 248 | 113 | 190 | 124 | 140 | 2480 | 3579 | 1771 | 1627 | 1412 | 2250 |
| Inorganic pyrophosphatase* | 1199 | 859 | 934 | 1061 | 1183 | 1755 | 1386 | 888 | 1123 | 1287 | 1057 | 1041 | 1056 | 921 | 958 | 18209 | 14654 | 17992 | 17657 | 14744 | 16144 |
| Invertase | 1 | 2 | 7 | 1 | 1 | 1 | 1 | 1 | 1 | 1 | 0 | 1 | 2 | 1 | 2 | 1 | 8 | 0 | 1 | 1 | 1 |
| Isoamylase* | 296 | 304 | 274 | 372 | 505 | 225 | 302 | 304 | 319 | 446 | 217 | 330 | 395 | 391 | 596 | 8002 | 7812 | 7439 | 9530 | 5780 | 9332 |
| Phosphofructokinase* | 49 | 50 | 163 | 76 | 98 | 70 | 79 | 62 | 72 | 32 | 83 | 66 | 68 | 49 | 78 | 1404 | 2219 | 868 | 1430 | 493 | 934 |
| Phosphoglucomutase, chloroplast, UniRef100_Q9M4G5 Cluster | 133 | 124 | 106 | 136 | 99 | 147 | 93 | 140 | 113 | 117 | 53 | 12 | 22 | 18 | 35 | 8397 | 5370 | 4749 | 6193 | 1518 | 1378 |
| Phosphoglucomutase, cytoplasmic, UniRef100_Q9M4G4 Cluster | 379 | 281 | 196 | 186 | 301 | 349 | 217 | 253 | 266 | 217 | 338 | 155 | 123 | 140 | 318 | 4335 | 4706 | 4117 | 5401 | 3410 | 5477 |
| Plastidic ATP/ADP-transporter | 1935 | 2874 | 2734 | 2177 | 2174 | 1361 | 1491 | 3443 | 1793 | 1941 | 1336 | 2968 | 1728 | 1350 | 2193 | 127665 | 87204 | 108384 | 115660 | 74164 | 83333 |
| Starch branching enzyme* | 3022 | 2509 | 2012 | 2082 | 1212 | 1896 | 1546 | 351 | 638 | 1109 | 1315 | 1149 | 1396 | 916 | 1135 | 75171 | 50551 | 51787 | 30196 | 40620 | 30806 |
| Starch synthase* | 1207 | 1359 | 1207 | 1224 | 1411 | 847 | 866 | 401 | 445 | 625 | 801 | 615 | 757 | 599 | 733 | 37171 | 33004 | 25487 | 18833 | 23847 | 37025 |
| Sucrose synthase* | 4623 | 2968 | 1645 | 2501 | 1576 | 5913 | 4830 | 970 | 1581 | 2183 | 3813 | 666 | 1111 | 914 | 1746 | 77803 | 36639 | 62752 | 30366 | 53601 | 37827 |
| Sucrose-phosphatase* | 106 | 198 | 174 | 161 | 131 | 111 | 137 | 81 | 78 | 212 | 60 | 63 | 84 | 81 | 104 | 3026 | 3613 | 1874 | 1652 | 1202 | 3349 |
| Sucrose-phosphate-synthase* | 1163 | 831 | 986 | 834 | 833 | 773 | 680 | 555 | 579 | 602 | 821 | 457 | 629 | 429 | 602 | 15270 | 18893 | 11466 | 15677 | 8375 | 15000 |
| UDP-glucose dehydrogenase 2 | 402 | 50 | 36 | 74 | 40 | 474 | 378 | 72 | 20 | 18 | 200 | 84 | 56 | 172 | 164 | 1680 | 2114 | 1813 | 3156 | 1339 | 1918 |
| UTP--glucose-1-phosphate uridylyltransferase | 3432 | 2624 | 2240 | 1928 | 1784 | 1384 | 1066 | 640 | 746 | 916 | 1796 | 1164 | 1526 | 1922 | 1488 | 15770 | 14886 | 8665 | 4938 | 11752 | 15684 |
